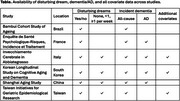# Associations between disturbing dreams and incident dementia and Alzheimer's disease across diverse international cohorts from the COSMIC collaboration

**DOI:** 10.1002/alz70857_096781

**Published:** 2025-12-24

**Authors:** Darren M. Lipnicki, Ashleigh S. Vella, Erico Costa, Karen Ritchie, Elena Rolandi, Ki Woong Kim, Ding Ding, Yen‐Ching Chen, Jen‐Hau Chen, Perminder S. Sachdev, Cohort Studies of Memory in an International Consortium (COSMIC)

**Affiliations:** ^1^ Centre for Healthy Brain Ageing (CHeBA), UNSW Sydney, Sydney, NSW, Australia; ^2^ René Rachou Institute, Fiocruz Minas, Belo Horizonte, Minas Gerais, Brazil; ^3^ Institut for Neurosciences of Montpellier, University Montpellier, National Institute for Health and Medical Research, Montpellier, France; ^4^ Golgi Cenci Foundation, Abbiategrasso, Milan, Italy; ^5^ Seoul National University Bundang Hospital, Seongnam, Korea, Republic of (South); ^6^ Institute of Neurology, Huashan Hospital, Fudan University, Shanghai, China; ^7^ Institute of Epidemiology and Preventive Medicine, College of Public Health, National Taiwan University, Taipei, Taiwan; ^8^ National Taiwan University Hospital, Taipei, Yunlin, Taiwan

## Abstract

**Background:**

Distressing dreams were reported to predict future dementia among predominantly white US participants aged 79‐89 years, particularly in men (Otaiku AI. eClinicalMedicine 2022;52:101640). We extended this research by investigating whether disturbing dreams (nightmares and bad dreams) predicted Alzheimer's disease (AD) additionally to all‐cause dementia among individuals aged more broadly (60‐89 years) and from diverse international regions.

**Method:**

Six longitudinal cohort studies from Brazil, China, France, Italy, South Korea, and Taiwan had complete data for 8339 participants (56.4% women) without baseline dementia or Parkinson's disease. Four studies had dementia/AD diagnoses, and 2 studies classified dementia using screening tests (Table). Disturbing dream data were harmonized as yes/no across all studies and weekly frequency (none, <1, ≥1) in 4 studies (3 with AD data, Table). All studies had a 21‐covariate set including sleep problems and medications, depression, and APOE*4. The maximum follow‐up across studies was 5.7‐16.6 years. Cox regressions with a random effect for study were used with all participants and stratified separately by sex and baseline age (60‐69, 70‐79, 80‐89 years). Analyses were repeated in study subsets with additional covariates, including anxiety (Table).

**Result:**

Disturbing dreams were reported by 24.3% overall. Dementia and AD incidence was 9.52 and 6.12 per 1000 person‐years, respectively. When including all age groups, disturbing dreams did not predict incident dementia, overall or in either sex. Similarly, disturbing dreams did not predict AD overall or in women, but any and ≥1/week disturbing dreams predicted AD among men in 2 fully‐adjusted studies (OR=2.03, *p* = .047; OR=4.00, *p* = .004). Age‐stratified analyses found effects only for 60‐69‐year‐olds. Among these, disturbing dreams predicted dementia across all studies (OR=1.89, *p* = .009) and 3 fully‐adjusted studies (OR=3.93, *p* = .014), and tended to predict AD in 2 fully‐adjusted studies (OR=4.96, *p* = .054). Additionally, disturbing dreams <1/week predicted dementia across 4 studies (OR=6.41, *p* < .001) and 3 fully‐adjusted studies (OR=4.65, *p* = .007), as well as AD in 3 studies (OR=4.94, *p* = .039) and 2 fully‐adjusted studies (OR=6.22, *p* = .031).

**Conclusion:**

Disturbing dreams predicted incident dementia and AD among individuals aged 60‐69 years, and incident AD among men aged 60‐89 years. Disturbing dreams may be a pre‐clinical indicator and possible prodrome of dementia and AD.